# Rehabilitating Long Edentulous Span by Using Pier Abutment as a Non-rigid Connector: A Case Report

**DOI:** 10.7759/cureus.51652

**Published:** 2024-01-04

**Authors:** Prasanna R Sonar, Aarati S Panchbhai, Ankita Pathak, Arushi Beri, Ravina Khairkar

**Affiliations:** 1 Oral Medicine and Radiology, Sharad Pawar Dental College and Hospital, Datta Meghe Institute of Higher Education and Research (DU), Wardha, IND; 2 Dentistry, Sharad Pawar Dental College and Hospital, Datta Meghe Institute of Higher Education and Research (DU), Wardha, IND; 3 Prosthodontics, Sharad Pawar Dental College and Hospital, Datta Meghe Institute of Higher Education and Research (DU), Wardha, IND; 4 Prosthodontics, Swargiya Dadasaheb Kalmegh Smruti Dental College and Hospital, Nagpur, IND

**Keywords:** non-rigid connector, long span edentulous arch, stress breaker, fixed dental prosthesis, pier abutment

## Abstract

An abutment having edentulous space on both sides is referred to as a pier abutment. The rehabilitation of a patient whose primary concern when they first came to the hospital was missing teeth is discussed in this case report. When using a pier abutment for a fixed dental prosthesis, there is a problem with fulcrum and torqueing pressures at the intermediate abutment. Forces on the pier abutment may become localized as a result of using a rigid connector. It has been suggested that non-rigid connectors be used to overcome this. Here, using a non-rigid connector as a stress breaker resulted in less stress being placed on the prosthetic assembly and abutment. This article offers a clinical case report that details the use of a non-rigid connector in a pier abutment scenario for rehabilitation.

## Introduction

In clinical practice, some concerns range from tooth movement and tooth loss to severe maxilla and mandibular fractures. However, some of the damaged dentition can be preserved using a prosthesis to stabilize the dentition and splint the abutment teeth. A fixed partial denture (FPD) splint is one possible treatment option for these patients.

When using a fixed dental prosthesis to replace lost teeth, biomechanical considerations must be carefully taken into account during prosthesis design. Connectors are the sections of an FPD or splint that attach the separate retainers and pontics [1,2]. Depending on the flexibility or movement at the connector joint, they can be either rigid or non-rigid. Rigid connectors, the most popular kind of connection used in FPDs, are advised for small unit FPDs with a single path of insertion [1]. In contrast, non-rigid connectors permit the movement of two components around one another, especially in a vertical plane [3]. It is recommended for mandibular arches with canine replacement, FPDs with misaligned abutments, abutments with an unfavorable prognosis, long-span bridge stress relief, and situations where the abutments' retentive capacities differ [3].

When a fixed prosthesis is necessary, it is not always advisable to choose an entirely rigid replacement [4]. A solo pier abutment will come up if there is an edentulous area adjacent to a tooth. Because of the teeth's mobility, the abutment location inside the arch, and the retainers' varying retention capacities, adopting an FPD is not advised [2,5].

By providing a mechanical union between the retainer and pontic that breaks the tension, non-rigid connections shift the stress from the connectors to the supporting bone. The types of non-rigid connectors are cross-pin, wing-type, dovetail-type, loop-type, and split-type connectors [6,7]. While allowing the abutments to move independently, a non-rigid fixed dental prosthesis appears to reduce mesiodistal torquing of the abutments [8]. This article uses a prefabricated resin connector pattern to create a long-span FPD with a pier abutment.

## Case presentation

**Figure 1:**
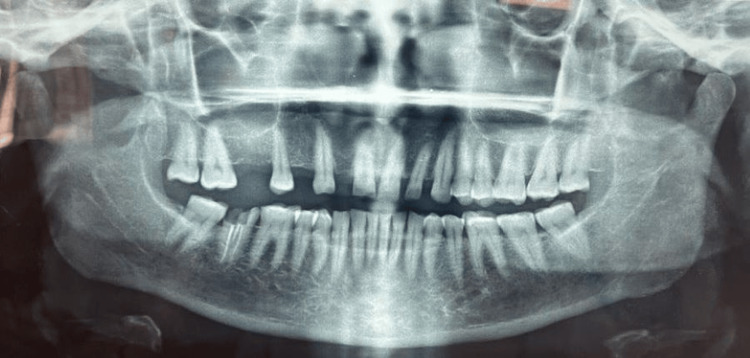
Orthopantomogram Image Credit: Prasanna Sonar

A 57-year-old woman presented with difficulties in masticating for five to six months due to several missing teeth in the upper right side of her jaw when she came to the dental hospital. Previous dental records showed that the first premolar, first molar, and right maxillary lateral incisor had all been extracted. Previous medical history was irrelevant. The second premolar is suitable for pier abutments. According to radiographic evaluation, teeth 13, 15, 17, and 18 were in good condition with a suitable crown-to-root ratio and no need for root canal therapy. Figure 1 shows a panoramic scanning of the upper and lower jaw. The three available treatment choices were FPD with a non-rigid connector, FPD with a rigid connector, and implant-retained prosthesis. Implant-retained dental prostheses were not considered since the patient refused to undergo surgery. In the long run, the patient's rehabilitation would involve the use of a porcelain-fused metal FPD with a non-rigid connector. Following a thorough evaluation and treatment planning, it was determined to provide non-rigid pier abutments on 15 teeth with cantilever prosthesis in the lateral incisor region. Simultaneously, rehabilitation of 47 was also advised for efficient mastication. However, the patient did not give consent for the same. Therefore, rehabilitation of the 12-17 regions was initiated.

**Figure 2:**
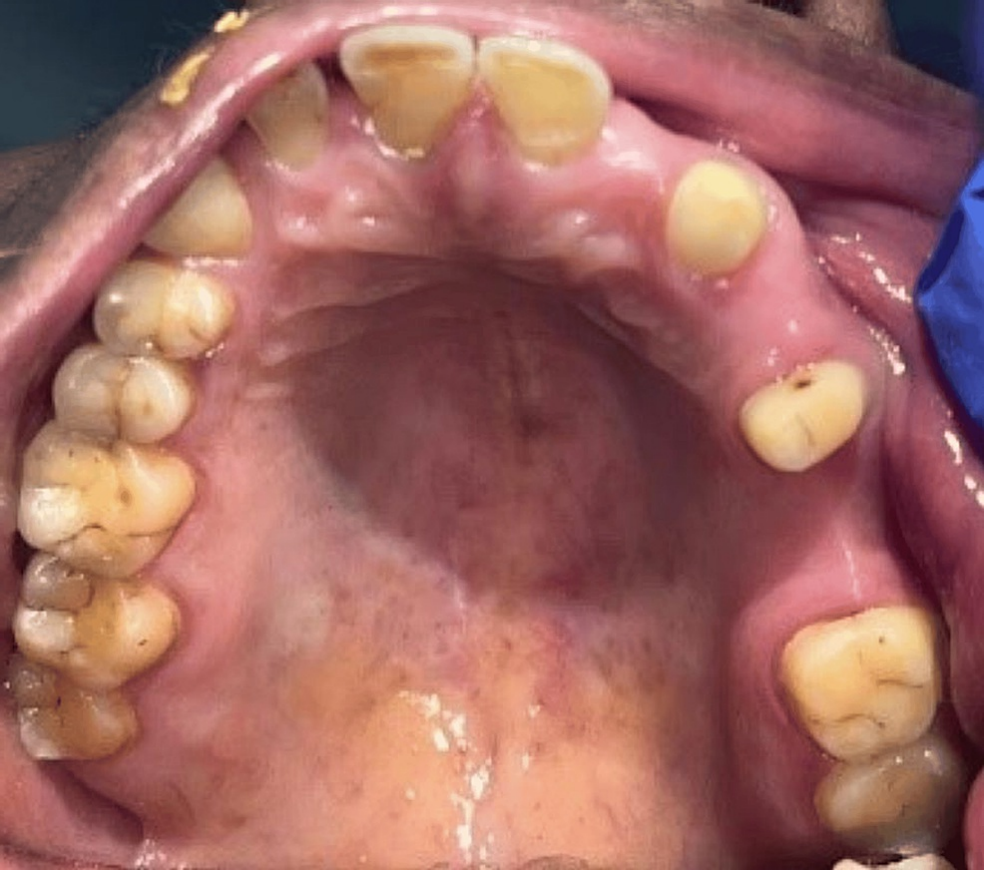
Teeth preparation Image Credit: Prasanna Sonar

A diagnostic wax and model were created. As seen in Figure 2, the maxillary right canine, second premolar, and second molar were prepared to receive porcelain-fused metal restorations with a subgingival margin and shoulder finish line.

**Figure 3:**
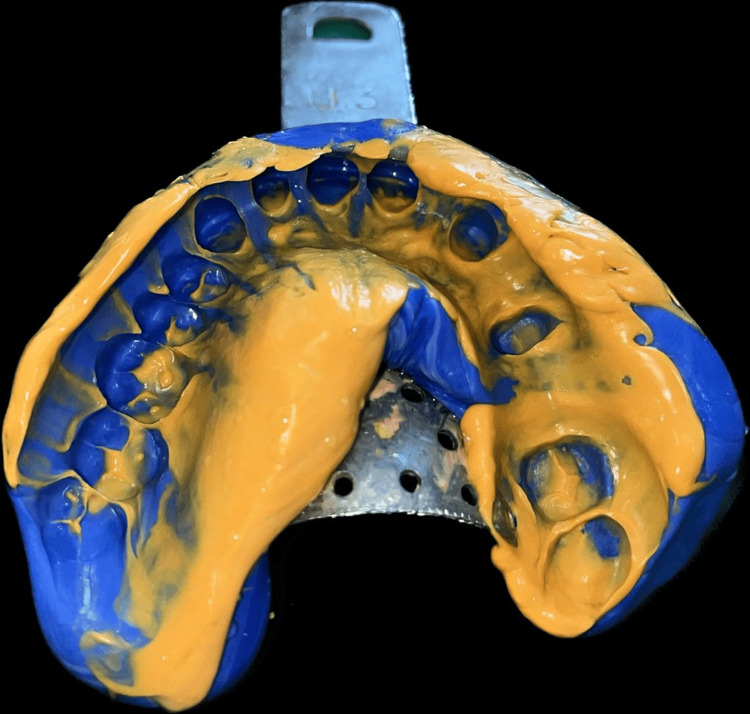
Final impression of the prepared teeth Image Credit: Prasanna Sonar

An elastomeric impression material was used to create the final impression, which was then poured into a die stone using a two-stage putty-wash procedure to create the master cast. The prepared teeth's final impression is shown in Figure 3.

**Figure 4:**
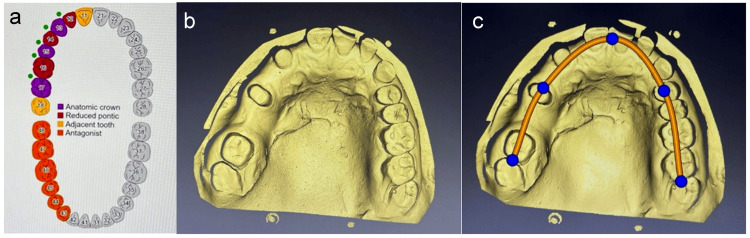
a) Data feeding into the software. b) Scanned image of the master cast. c) Occlusal plane marked on the cast in the software Image Credit: Prasanna Sonar

To create an interocclusal record, bite registration material was employed. A tooth-colored auto-polymerizing acrylic resin was used to create the temporary restorations, which were then sealed using non-eugenol temporary cement. Exocad software was used to scan and design the cast, as shown in Figure 4. Figure 4a shows data feeding into the software, Figure 4b shows a scanned image of the master cast, and Figure 4c shows an occlusal plane marked on the cast in the software.

**Figure 5:**
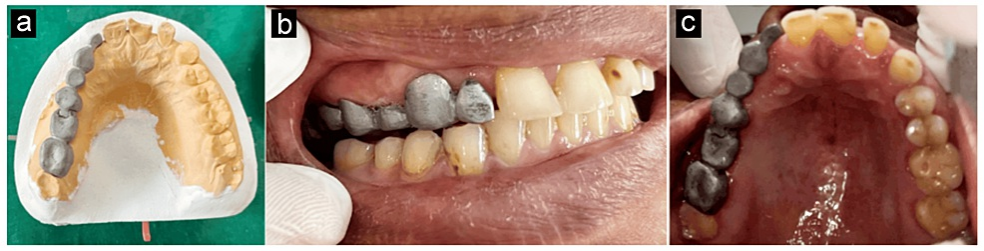
a) Metal try-in on cast. b) Metal try-in on patient (lateral view). c) Metal try-in on patient (occlusal view) Image Credit: Prasanna Sonar

A milling machine was used to mill the wax design. By surveying, it was determined that the plastic male attachment was parallel. Casting and investing were finished. Using an elastomeric impression material, a pickup imprint was created, and the patient had a metal anterior attachment try-in using a keyway or mortise. Casting techniques were applied. After inserting the male and female components, the metal fit was assessed in the lab, as depicted in Figure 5a. Clinical testing was performed on both the front and posterior sections, as shown in Figure 5b and Figure 5c, to verify that the restoration's marginal fit was suitable.

**Figure 6:**
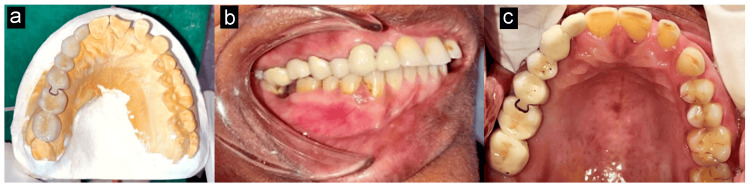
a) Trial in the working cast. b) Final prosthesis cemented with GIC (lateral view). c) Final prosthesis cemented with GIC (occlusal view) GIC: glass ionomer cement Image Credit: Prasanna Sonar

It was caramelized after the shade was selected. The anterior segment was joined to the female component (keyway mortise) and the posterior segment to the male portion (key tenon) in the working cast, as illustrated in Figure 6a, to complete the laboratory method. As demonstrated in Figure 6b and Figure 6c, glass ionomer cement (GIC) was used to cement the prosthesis. Following the removal of excess cement, the occlusion was examined using an articulating paper. The patient received instruction on maintaining good oral hygiene, including using floss, and interdental brush was recommended. The patient was inspired to understand the value of routine follow-up appointments. After a month, the patient was evaluated and advised for a further follow-up visit and rehabilitation of 47.

As the rehabilitation of 47 was not done, the efficiency of mastication was not adequate. This is the limitation of this case report.

## Discussion

Fixed dental prostheses typically use rigid connectors due to their ease of fabrication. On the other hand, a scenario with pier abutments presents a challenging situation in which the central pier abutment serves as a pivot. A non-rigid connector must serve as a stress breaker in this scenario since a rigid connector in a pier abutment FPD causes higher rates of debonding due to the dislodging pressures. Non-rigid connectors, however, should only be used sparingly and should not be recommended in cases where the abutment exhibits significant mobility, where the edentulous span on either side of the pier abutment is greater than one tooth, or when the FPD with non-rigid connector is opposed by a combination of edentulous ridge/removable prosthesis and natural teeth/fixed prosthesis [9-14].

The stress-relieving action or stress-breaking effect of non-rigid connections shields and keeps the abutment teeth from breaking. On the other hand, flexion in the mesiodistal direction caused by splinting with a rigid connection results in the failure of the fixed prosthesis because of the load on the surface of the abutment tooth [15]. When a pier abutment is present, force is applied to the terminal retainers, causing them to encroach during function. The middle retainer acts as a fulcrum, causing the weaker retainer to fail [15]. This can be avoided in a five-unit fixed prosthesis by utilizing a non-rigid pier connector that distributes stress as little as possible in the pier abutment [16].

The secret to a successful FPD is selecting the correct connectors. The following are indications for non-rigid connectors [9]: Pier abutment intrusion and the failure of the weakest terminal abutments can occur as a result of the fulcrum-like condition created by the presence of pier abutments. The presence of the misaligned abutment, where devitalization could occur from parallel preparation, is another indication for non-rigid connectors. Also, long-span FPD could change how the prosthesis fits against the teeth because of porcelain traction and thin areas of the framework contracting. When the jaw opens and closes, it extends mediolaterally, indicating a non-rigid link in the anterior and posterior parts of the mandibular arch, or FPD. Another indication is the variation in the abutments' ability to retain material. The following are non-rigid connector contraindications [17]: presence of significant mobility in the abutment and destructive pressures could be transferred to the abutment underneath the soldered retainer if there is a space between the abutments that is greater than a single tooth. Regarding the location of connectors which are non-rigid type, opinions differ.

Shillingburg et al. advised positioning the non-rigid connector on the middle abutment in the five-unit pier abutment restoration since positioning it on either of the terminal abutments could cause the pontic to act as a lever arm. The key must be placed on the medial side of the distal pontic, and the connector's keyway must lie inside the typical distal contours of the pier abutment. The posterior teeth's long axes typically slant somewhat mesially, and additional movement in this direction is brought about by vertically applied occlusal forces. If the key is positioned on the distal side of the pier abutment, mesial movements will seat it more firmly into the connector's keyway [16]. This position is further supported by the finite element research of Oruc et al. [16], which indicated that the adoption of non-rigid connectors in the distal region of the second premolar decreased the area of maximum stress concentration in pier abutments. Markley advised against installing the non-rigid connector at the pier abutment because it would place too much stress on the premolar abutment, which is comparatively weak [18]. Gill recommended installing a non-rigid link on the pier abutment on one or both sides [19]. Adams advised placing a non-rigid connector to the pier's distal side and, if preferred, inserting an additional one to the anterior retainer's distal side [20].

## Conclusions

The future success of an FPD is significantly influenced by the size, shape, and kind of connectors. An essential first step in the treatment planning of a pier abutment is choosing the appropriate connector. Non-rigid connectors permit physiologic tooth movement while simultaneously transferring less stress to abutments. By neutralizing all pressures acting on the connector, the non-rigid connectors serve as stress breakers, safeguarding the abutment teeth. It prolongs the life of the restoration and offers a safety valve system for long-span bridges. A viable option for enhancing the durability, stability, and use of prosthetic restorations is the integration of non-rigid connectors into FPDs. This case study highlights the significance of careful assessment, accurate execution, and ongoing supervision to maximize the effectiveness of non-rigid connector-based FPDs in restorative dentistry, even when they present positive results.
